# Understanding the Battle for Universal Pharmacare in Canada Comment on "Universal Pharmacare in Canada"

**DOI:** 10.34172/ijhpm.2020.40

**Published:** 2020-03-14

**Authors:** Marc-André Gagnon

**Affiliations:** School of Public Policy and Administration, Carleton University, Ottawa, ON, Canada.

**Keywords:** Universal Pharmacare, Canada, Catastrophic Coverage, Health Insurance, Pharmaceuticals

## Abstract

Drug coverage in Canada is a patchwork; an inequitable inefficient and unsustainable patchwork with no coherence or purpose. Some people think that we can solve the problem by adding more patches, but the core of the problem is that it is a patchwork. For the working population, access to medicines is still organized as privileges offered by employers to their employees. Universal pharmacare would not only provide better access to needed prescription drugs, but also eliminate waste, ensure value-for-money and help improve drug safety and appropriate prescribing. Opponents fear that a universal pharmacare plan would ration drugs, and impede drug access for some patients. However, these claims misunderstand the reality of drug coverage, pricing and access. Opponents propose, instead, to "fill the gap" of current drug coverage by implementing catastrophic coverage, which would serve commercial interests without maximizing health outcomes for the Canadian population. In spite of overwhelming evidence and consensus in the academic community in favour of universal pharmacare, the battle is far from over.

## Introduction

Following the publication in Canada of a landmark report published by the *Advisory Council for the Implementation of National Pharmacare* (ACINP) calling for the implementation of a single-payer universal Pharmacare,^1^ two recent *IJHPM* articles discussed drug coverage reform in Canada. Hajizadeh and Edmonds^2^ explained why Canada should implement universal pharmacare for equity reasons while Lewis^3^ explained how difficult this implementation would be due to important vested interests that benefit from the current expensive and wasteful system. This commentary explains why the current system needs to change and explores the two options on the table: universal pharmacare versus catastrophic coverage.

## An Inefficient and Inequitable System

Canada is the only country that offers universal healthcare coverage without covering prescription drugs, as if it were not an essential healthcare service.^4^ For the working population and their dependents, access to medicines is still organized as privileges offered by employers to their employees. Meanwhile, the non-working population is covered by a patchwork of public plans where copays, deductibles, and the list of drugs covered depends on the province or territory on which they live. The outcomes are problematic. Overall, 18% of Canadian households face catastrophic out-of-pocket expenses on pharmaceutical products,^2^ and around 10% of Canadians declared not having filled a prescription or skipped doses in the last 12 months due to financial reasons.^5^ While many Canadians cannot access the drugs they need, Canadians spend on average 43% more per capita on retail pharmaceuticals than the median of Organisation for Economic Co-operation and Development countries,^6^ mostly due to the fact that Canadian drug plans pay higher prices for the same drugs and are less effective in implementing cost-containment measures.^7^

Canada currently has over 100 public drug plans (mostly provided by the provinces and territories to cover the non-working population) and more than 100 000 private drug plans provided by employers, all with their set of premiums, copayments, deductibles and annual limits.^1^ Furthermore, the purpose of private drug plans has more to do with pleasing employees during collective bargaining than to maximize health outcomes through evidence-based coverage.^8^

The majority of private plans accept covering any drug at any price as soon as it is approved by Health Canada.^9^ For example, the antidepressant Trintellix (vortioxetine) does not demonstrate any additional benefits in contrast to similar products, such as escitalopram, that cost 10 times less.^10^ However, it is still covered by most private plans, and the drug managed to capture 9% of the Canadian market for depression.^11^ Similarly, 88% of the new patented drugs that entered the Canadian market in 2017 did not represent a significant therapeutic improvement as compared to existing products,^12^ and many of these drugs, like Trintellix, are priced much higher than comparable products. Due to the fact that the Canadian market for prescription drugs does not apply efficient standards to ensure value for money, a significant portion of drug spending goes to waste while many Canadians are struggling to access the drugs they need.

Drug coverage in Canada is a patchwork; an inequitable, inefficient, and unsustainable patchwork with no coherence or purpose. Some people think that we can solve the problem by adding more patches, but the core of the problem is that it is a patchwork. In the Canadian fragmented system, stakeholders end up with little incentive to invest in institutional capacities to support prescribers (or patients) in maximizing best practices and appropriate prescribing to improve health outcomes, because they would foot the bill while reaping only a fraction of the benefit. One of the first ACINP recommendations was to invest in drug-related information technology to start collecting data and monitoring prescribing habits.^13^ In the current system, we do not even have these basic institutional tools that would be critical to deal with issues such as the current opioid crisis, which stems from irrational prescribing.

Universal pharmacare would not only provide better access to needed prescription drugs, it would also reduce waste, increase value-for-money, and help improve drug safety and appropriate prescribing. Following the publication of the ACINP report, 1300 Canadian academic experts in healthcare and public policy called for policy-makers to implement the report’s recommendations.^14^

## A Well-Organized Resistance

It is estimated that universal pharmacare would save Canadians between 20% and 40% of current expenditures on prescription drugs^7,15,16^ by reducing waste and developing bargaining capacity to negotiate lower drug prices. However, every expenditure is somebody else’s income; thus considerable savings for some means considerable losses for others. Some stakeholders face losing billions and are deploying substantial means to make sure that it does not happen. For example, GlaxoSmithKline disclosed its financing of a National Pharmacare Advocacy Program organized with patient groups willing to stand against universal pharmacare.^17^ Also, most “analyses” opposing universal pharmacare are published through the *Canadian Health Policy Institute,* a think tank created and managed by an executive director of Innovative Medicines Canada, the lobby group of drug companies. The organization publishes a fake peer-reviewed journal in which one-sided analyses that would never be accepted in any peer-reviewed academic journal can easily be published. These one-sided studies can then be cited in the academic literature in a way that looks legitimate, or can serve as the basis to secure op-eds in Canadian media.^18^

The main claims of those opposing universal pharmacare is that such drug coverage reform would eliminate “more generous” private drug plans and reduce current access Canadians have to more expensive drugs.^19,20^ Most of these claims are simply misleading; the fear that a universal pharmacare plan would ration drugs, and impede drug access for some patients, misunderstands the reality of drug coverage, pricing, and access.

If universal public coverage for cost-effective drugs was introduced in Canada based on the ACINP recommendations, this would not restrain employers from offering supplemental drug coverage. Savings on private benefits for drugs covered by a public plan would reduce labour costs, which would allow employers to offer additional health benefits, increase wages, or simply increase profits. Removing current coverage offered by employers is not on the radar and employers could maintain, at lower cost, the current level of drug benefits they offer through supplemental coverage.

Then why all the fuss? One issue is that employer-provided drug benefits are not considered income, so it is not taxable and private plans might thus lose an important advantage. Our governments pay billions each year in tax subsidies for private drug benefits as an incentive for employers to provide private drug coverage. Such tax subsidies, however, mean that the more income you gain, the more subsidies you get, and the less incentive you have for things to change. The other issue is that if Canadians have a good public drug plan, some employers might consider excluding supplemental coverage for drugs that are not cost-effective and offering other types of benefits instead. This is where things get a bit awkward: some stakeholders are convinced that public universal pharmacare is not “generous” enough as compared to current private plans, but they are also convinced that employers would have no interest in offering supplemental benefits if a good public drug plan exists. Instead of making the case to employers that they should offer supplemental coverage over public drug coverage because it would benefit employers and employees, these stakeholders prefer to fight against universal public coverage to force employers to fully provide wasteful drug benefits.

## Catastrophic Coverage for Dummies

Opponents of universal pharmacare agree that there are issues with access to medicines, but call for “filling the gap”^21^ with what is called catastrophic coverage.^22,23^ For example, once an employee (or their employer) pays more than 3% of the employee’s annual income in prescription drugs, the additional bill would be covered by the public drug plan. This logic needs to be explored. Private plans have been hit hard by rising costs of specialty drugs.^24^ Employees (or their dependents) requiring expensive drugs are prime examples of “bad risks” for an employer-sponsored drug plan. By definition, private insurance always tries to insure only “good risks” and get rid of “bad risks.”^25^ Considering that risks are pooled (and premiums are set) in employer-sponsored drug insurance based on the workplace, imagine what it would mean for the drug plan of a workplace with 100 employees if, for example, the daughter of an employee is diagnosed with a rare disease that requires a drug costing $300 000/year. If the employer accepts to reimburse the drug, it means that premiums for each employee will increase by $3000/year. To avoid this, private plans are protecting themselves by implementing annual caps on reimbursement, or by refusing to cover more expensive drugs.^25^ Another solution for the employer is to simply lay off the “bad risk employee.” In comparison, a public drug plan organized at a populational level and using best available evidence for health technology assessment would not only pool risks on the whole population, but could use outcomes-based risk sharing agreements to cover very expensive drugs while still getting value for money.^1,26^

However, the ideal solution for private insurers would be to have the possibility to simply dump “bad risks employees” onto a public plan. This is what catastrophic coverage is about. In a nutshell, the position of private insurers would be to maintain a highly subsidized, inequitable, and inefficient system for the private coverage of “non-risks.” This is not health insurance, this is institutionalized corporate welfare. Public drug coverage must be organized to maximize the health outcomes for all Canadians in a cost-effective way, not as a “risk dump” to serve commercial interests.

## Conclusion

In spite of overwhelming evidence and consensus in the academic community in favour of universal pharmacare, the battle is far from over. Olson^27^ once explained that when a large population has a common interest in achieving collective action, this common interest can be supplanted by minority groups bound together by concentrated selective incentive. In the case of drug coverage, there is a lot of money at stake and potential losers like drug companies, insurance companies, and pharmacy chains that are very well organized and are known for their powerful lobbying.^18^ When I started working on drug coverage reforms 10 years ago,^28^ and saw how Canadians would benefit while saving billions with universal pharmacare, I thought the case was closed and political reform would naturally happen. Ten years later, I realize that it might be because of the magnitude of these savings and the opposition it creates from vested interests that universal pharmacare might not happen in Canada. The battle is still raging and, in some way, my personal faith in democracy is at stake.

## Ethical issues

Not applicable.

## Competing interests

I have no relationships with commercial interests. My current research benefited by funding received from public sources or NGOs including a Canadian Institutes for Health Research Catalyst grant on institutional corruption in the pharmaceutical sector; a Health Canada research contract on funding national drug coverage; funding from the WHO Collaborating Center on Governance, transparency and accountability in the pharmaceutical sector. I also received honoraria for presentations on drug prices and drug coverage from Canadian Association of Pharmacy in Oncology; Association des Neurologues du Québec, Syndicat Québécois des employé(e)s de service, Fédération des travailleurs et travailleuses du Québec, Syndicat canadien de la fonction publique and Centrale des syndicats démocratiques. I also acted as an Expert witness for Justice Canada in a lawsuit over Canadian drug price regulation involving drug companies.

## Author’s contribution

MAG is the single author of the paper.
